# Trends and Factors Associated With Physician Burnout at a Multispecialty Academic Faculty Practice Organization

**DOI:** 10.1001/jamanetworkopen.2019.0554

**Published:** 2019-03-15

**Authors:** Marcela G. del Carmen, John Herman, Sandhya Rao, Michael K. Hidrue, David Ting, Sara R. Lehrhoff, Sarah Lenz, James Heffernan, Timothy G. Ferris

**Affiliations:** 1 Massachusetts General Physicians Organization, Boston; 2 Department of Obstetrics, Gynecology and Reproductive Biology, Massachusetts General Hospital, Harvard Medical School, Boston; 3 Department of Psychiatry, Massachusetts General Hospital, Harvard Medical School, Boston; 4 Partners Health, Boston, Massachusetts; 5 Department of Medicine, Massachusetts General Hospital, Harvard Medical School, Boston

## Abstract

**Importance:**

Physician burnout is common, and prevalence may differ throughout a clinician’s career. Burnout has negative consequences for physician wellness, patient care, and the health care system. Identifying factors associated with burnout is critical in designing and implementing initiatives to reduce burnout.

**Objective:**

To measure trends and identify factors associated with physician burnout.

**Design, Setting, and Participants:**

Survey study conducted from May 16 to June 15, 2014, and again from May 16 to June 15, 2017, measuring rates of physician burnout in a large academic medical practice. Factors associated with burnout out were evaluated. In 2014, 1774 of 1850 eligible physicians (95.9%) completed the survey. In 2017, 1882 of 2031 (92.7%) completed the survey.

**Exposures:**

Medical specialty, demographic characteristics, years in practice, and reported rates of burnout.

**Main Outcomes and Measures:**

Burnout rates measured at 2 points and risk factors associated with burnout.

**Results:**

Respondents included 1027 men (57.9%) and 747 women (42.1%) in 2014 and 962 men (51.1%) and 759 women (40.3%) in 2017. The mean (SD) number of years since training completion was 15.3 (11.3) in the 2014 survey data and 15.1 (11.3) in the 2017 data. Burnout increased from 40.6% to 45.6% between the 2 points. The increased rate was associated with an increase in exhaustion (from 52.9% in 2014 to 57.7% in 2017; difference, 4.8%; 95% CI, 1.6%-8.0%; *P* = .004) and cynicism (from 44.8% in 2014 to 51.1% in 2017; difference, 6.3%; 95% CI, 3.1%-9.6%; *P* < .001). Compared with midcareer physicians (11-20 years since training), early-career physicians (≤10 years since training) were more susceptible to burnout (odds ratio, 1.36; 95% CI, 1.05-1.77), while physicians in their late career (>30 years since training) were less vulnerable (odds ratio, 0.59; 95% CI, 0.40-0.88).

**Conclusions and Relevance:**

Efforts to alleviate physician burnout and administrative burden require a combination of a shared commitment from physicians and organizations and central and locally implemented programs. Continued research is necessary to establish the most effective initiatives to decrease physician burnout at the individual and organizational level.

## Key Points

**Question:**

What trends and factors are associated with physician burnout in a large academic medical faculty practice?

**Findings:**

In this survey study administered to 1774 physicians in 2014 and 1882 physicians in 2017, burnout increased from 40.6% to 45.6%, with early-career physicians more likely to report burnout than midcareer and late-career physicians. An institution-wide process was mobilized to address and relieve burnout.

**Meaning:**

Relief of burnout will require local and central initiatives and engagement of both individuals and leadership.

## Teaser

This survey study assesses trends and factors associated with physician burnout in a large academic medical faculty practice.

## Introduction

Physician burnout has been described extensively in the peer-reviewed literature.^1,2,3,4^ Freudenberger^1^ first described the concept of staff burnout in terms of physical signs and behavioral indicators. Maslach et al^2^ defined burnout as a syndrome characterized by depersonalization, emotional exhaustion, and sense of low personal accomplishment.

Burnout rates among physicians vary by geographic location, experience level, specialty, age, and sex.^4^ It is estimated that at least 35% of physicians in the developing world and 50% in the United States experience burnout, now considered by many experts to be an epidemic.^4,5^ Burnout rates are higher for physicians engaged in the front lines of care, including family medicine, emergency medicine, and general neurology.^6,7,8^

Burnout carries significant professional and personal consequences.^6,7,8,9^ Physician burnout is negatively associated with altruism, professionalism, and quality and safety of care.^9,10,11^ At the individual level, physician burnout has been associated with cardiovascular disease, alcohol use, depression, suicide, and shorter life expectancy.^12,13,14,15,16,17,18,19^ Burnout has also been implicated as having an adverse financial impact on practices and organizations.^6^ Here, we present data on administrative burden from a hospital-wide physician practice survey conducted in 2017. When appropriate, we compare these data with those collected in 2014 using the same instrument and sampling the same population.

## Methods

### Survey Instrument

The Massachusetts General Physicians Organization conducts a biennial survey of active members of this multispecialty academic faculty practice. Qualifying physicians earn a financial incentive for completing the survey, which is part of the organization’s Quality Incentive Program.^20,21^ Eligibility for the incentive program is based on percentage of time physicians spend on clinical activity. Incentive amount for completing the survey ranges from $166.67 to $833.34 depending on the percentage of time the participant spends on clinical activity. Physicians are asked to consent to survey participation on the cover letter inviting them to participate. The survey, approved by the Partners human research committee, was most recently administered in 2017. It assesses physician perceptions of the functioning of the clinical enterprise within and across departments, reflects progress made on organizational priorities, and evaluates hospital leadership.^20,21^

The survey assesses 4 domains: (1) physician career and compensation satisfaction; (2) physician well-being (assessed using the Maslach Burnout Inventory and a 5-point Likert scale); (3) administrative workload on physicians; and (4) leadership and diversity content.^2^ The Maslach Burnout Inventory evaluates physician burnout by grouping items into 3 scales: exhaustion, cynicism, and professional efficacy.^2^ As described in the Maslach Burnout Inventory manual, a score of 3.0 or greater on the exhaustion subscale, 2.0 or greater on the cynicism subscale, or 4.0 or greater on the professional efficacy subscale denotes a high level of burnout for the respondent in that subscale.^2^ Many studies measuring physician burnout using the Maslach Burnout Inventory use the convention that burnout is determined by the percentage of physicians scoring high on either emotional exhaustion or cynicism. We elected to use the 3 domains because this was the original description of the tool. Given the concern that using the original version of the inventory would result in underreporting of our faculty group’s burnout rate, we calculated the data using both the 3 domains originally described in the Maslach Burnout Inventory and the revised version, using 2 domains and dropping professional efficacy. The results were similar, with a 5% increase noted in both calculations between 2014 and 2017. Career satisfaction is measured using a 5-point Likert scale (very satisfied, satisfied, neutral, dissatisfied, or very dissatisfied).^21^ All methods for both surveys are in compliance with the American Association for Public Opinion Research (AAPOR) reporting guideline for survey studies.^22^ We report and compare data from the 2017 and 2014 Massachusetts General Physicians Organization surveys.

### Statistical Analysis

To compare demographic distribution, level of burnout, and level of satisfaction between the 2014 and 2017 surveys, we used χ^2^ tests and *t* tests, as appropriate. We then used multivariable regression to evaluate the association of factors with selected outcomes. All statistical tests were 2-sided and *P* values less than .05 were considered statistically significant. Regression results are reported as odds ratios (ORs). All statistical analyses were performed using SAS statistical software version 9.4 (SAS Institute, Inc).

## Results

### Survey

A total of 1774 of 1850 (95.9%) and 1882 of 2031 (92.7%) of eligible physicians completed the 2014 and 2017 Massachusetts General Physicians Organization survey, respectively. Demographic distributions of respondents are presented in Table 1. Respondents included 1027 men (57.9%) and 747 women (42.1%) in 2014 and 962 men (51.1%) and 759 women (40.3%) in 2017, with 161 participants (8.5%) declining to specify their sex. The mean (SD) number of years since training completion was 15.3 (11.3) in the 2014 survey data and 15.1 (11.3) in the 2017 data.

**Table 1.** Demographic Distribution of the 2014 and 2017 Surveys

Demographic Characteristic	No. (%)		*P* Value
	2014 (n = 1774)	2017 (n = 1882)	
Sex^a^			
Male	1027 (57.9)	962 (51.1)	<.001
Female	747 (42.1)	759 (40.3)	.27
Did not specify	NA	161 (8.5)	
Race			
Asian	285 (16.1)	266 (14.1)	.10
White	1304 (73.5)	1334 (70.9)	.07
Other	185 (10.4)	282 (14.9)	<.001
Time since training, y			
≤10	768 (43.3)	819 (43.5)	.89
11-20	508 (28.6)	518 (27.5)	.45
21-30	297 (16.7)	332 (17.6)	.47
>30	201 (11.3)	213 (11.3)	.99
Specialty			
Emergency medicine, radiology, anesthesia, and pathology	357 (20.1)	336 (17.9)	.08
Medicine	985 (55.5)	1117 (59.4)	.02
Primary care physician	262 (14.9)	251 (13.3)	.18
Surgery	168 (9.5)	178 (9.5)	.99

Abbreviation: NA, not applicable.

^a^ The 2014 survey had 2 options for sex (male and female), while the 2017 had 3 options (male, female, and prefer not to say). The change in proportion from 2014 to 2017 likely reflects this change.

In 2017, 45.6% of physicians scored high in 2 of 3 of the Maslach burnout scales, compared with 40.6% in 2014 (Table 2). An increase in exhaustion (from 52.9% in 2014 to 57.7% in 2017; difference, 4.8%; 95% CI, 1.6%-8.0%; *P* = .004) and cynicism (from 44.8% in 2014 to 51.1% in 2017; difference, 6.3%; 95% CI, 3.1%-9.6%; *P* < .001) were associated with the increase. Most physicians were satisfied with the quality of care they provide (≥89%), relationship with their colleagues (≥84%), opportunities for consulting with their peers (≥85%), and their practice’s call and coverage schedule (≥71%). These results have not changed significantly over the 2 surveys. On other measures, we observed lower rates of satisfaction with modest improvements from 2014 to 2017. These include administrative support (from 30.4% to 34.8%; difference, 4.4%; 95% CI, 1.4%-7.5%; *P* = .004) and having a trusted advisor (from 46.7% to 49.7%; difference, 3.0%; 95% CI, −0.2% to 6.2%; *P* = .07). Work satisfaction decreased between the 2 surveys. Measures included satisfaction with workload (from 55.0% to 50.5%; difference, −4.5%; 95% CI, −7.8% to −1.3%; *P* = .006), control over one’s schedule (from 71.6% to 64.3%; difference, −7.4%; 95% CI, −10.4% to −4.3%; *P* < .001), and opportunity to have an impact on decision-making processes affecting day-to-day clinical practice (from 58.7% to 55.4%; difference, −3.3%; 95% CI, −6.5% to −0.1%; *P* = .004). Finally, the average time spent by a physician on administrative duties increased from 23.7% in 2014 to 27.9% in 2017 (difference, 4.2%; 95% CI, 3.0%-5.3%; *P* < .001) (Table 2). The top 3 duties requiring more faculty engagement between the 2 survey periods included time spent on prior authorizations, time spent on specialty recertification, and time spent on compliance regulations related to opioid prescribing.

**Table 2.** Comparing Burnout and Satisfaction With Selected Domains of Physician Work Between 2014 and 2017 Surveys

Survey Results	No. (%)		*P* Value
	2014 (n = 1774)	2017 (n = 1882)	
Burnout^a^			
High exhaustion	938 (52.9)	1085 (57.7)	.004
High cynicism	794 (44.8)	962 (51.1)	<.001
Low professional efficacy	318 (17.9)	373 (19.8)	.14
Experienced burnout (high in 2 of 3 scales)	720 (40.6)	859 (45.6)	.002
Satisfied with^b^			
Quality of care you provide	1602 (90.3)	1680 (89.3)	.30
Relationship with colleagues	1502 (84.7)	1624 (86.3)	.16
Opportunities to consult with peers	1530 (86.2)	1615 (85.8)	.70
Your practice’s call and coverage schedule	1306 (73.6)	1337 (71.0)	.08
Time and resources provided for continuing medical education	995 (56.1)	1010 (53.7)	.14
Your practice’s workflow	967 (54.5)	985 (52.3)	.18
Control over your schedule	1271 (71.6)	1210 (64.3)	<.001
Opportunity to impact decision making that affects your day-to-day clinical practice	1041 (58.7)	1043 (55.4)	.004
Your workload	976 (55.0)	950 (50.5)	.006
Agreed with^c^			
I have a trusted advisor	829 (46.7)	936 (49.7)	.07
I have enough administrative support	539 (30.4)	655 (34.8)	.004
Administrative duties affect my ability to deliver quality care	1145 (64.5)	1170 (62.2)	.13
Time spent on administrative duty, mean (SD), min	278 (23.7)	525 (27.9)	<.001

^a^ The following cutoffs are used for defining a high score for each subscale of burnout: exhaustion, 3.0 or greater; cynicism, 2.0 or greater; and professional efficacy, 4.0 or greater.

^b^ A 5-point Likert scale (very satisfied to very dissatisfied) was used to measure respondents’ satisfaction with different aspects of their work. Those who responded with very satisfied or satisfied are defined as satisfied.

^c^ A 5-point Likert scale (strongly agree to strongly disagree) was used to measure respondents’ agreement with these statements. Those who responded with strongly agree or agree are considered here as agreed.

Demographic factors associated with burnout, including sex, ethnicity/race, years of experience, and specialty, were evaluated using multivariate analyses. Work-related factors were also included in the assessment of variables deemed to be associated with burnout. Table 3 presents results of multivariate analysis predicting likelihood of burnout for both the 2014 and 2017 surveys. Primary care physicians practicing in the ambulatory setting were more likely to have exhaustion at both points when compared with medicine specialists (OR, 1.39; 95% CI, 1.005-1.92 in 2014 and OR, 1.42; 95% CI, 1.02-1.99 in 2017; *P* < .001). Although we highlight the 2017 survey results, most of the factors’ association with burnout remained stable on both surveys.

**Table 3.** Multivariate Logistic Regression Predicting Likelihood of Burnout^a^

Factor	Odds Ratio (95% CI)	
	2014 Survey	2017 Survey
Female (reference = male)	0.85 (0.68-1.07)	0.85 (0.67-1.07)
Race (reference = white)		
Asian	0.67 (0.49-0.92)	1.13 (0.83-1.54)
Other	0.90 (0.63-1.30)	1.06 (0.76-1.48)
Time since training (reference = 11-20 y), y		
≤10	1.40 (1.08-1.83)	1.36 (1.05-1.77)
21-30	0.78 (0.56-1.08)	0.84 (0.61-1.16)
>30	0.60 (0.40-0.89)	0.59 (0.40-0.88)
Specialty (reference = medicine)		
Emergency medicine, radiology, anesthesia, and pathology	1.08 (0.80-1.46)	1.02 (0.75-1.37)
Primary care physicians	1.39 (1.005-1.92)	1.42 (1.02-1.99)
Surgery	0.79 (0.54-1.18)	0.70 (0.48-1.04)
Career misfit	1.53 (1.07-2.18)	1.12 (0.78-1.60)
Satisfaction with^b^		
Relationship with colleagues	0.70 (0.50-0.98)	0.53 (0.38-0.75)
Quality of care	0.47 (0.31-0.70)	0.77 (0.52-1.13)
Workflow	0.68 (0.53-0.88)	0.62 (0.48-0.79)
Call and coverage schedule	0.75 (0.57-0.98)	0.85 (0.65-1.11)
Time and resources for continuing medical education	0.78 (0.61-0.99)	0.74 (0.59-0.92)
Opportunity to consult with peers	1.02 (0.73-1.43)	0.73 (0.52-1.01)
Control over schedule	0.81 (0.62-1.06)	0.89 (0.69-1.15)
Opportunity to impact decision making	0.72 (0.56-0.93)	0.73 (0.56-0.94)
Workload	0.56 (0.43-0.72)	0.47 (0.37-0.59)
Agreement with^b^		
Having a trusted advisor	0.58 (0.46-0.74)	0.67 (0.53-0.84)
Having enough administrative support	1.16 (0.89-1.50)	0.94 (0.73-1.19)
Administrative duty affects my ability to provide care	1.82 (1.42-2.34)	1.55 (1.23-1.96)
Time spent on administrative duty, min	1.01 (1.01-1.02)	1.01 (1.01-1.02)

^a^ Burnout was defined as a binary variable in which a high score on 2 of the 3 burnout scales is defined as 1 and 0 otherwise. An alternative analysis using a continuous specification of exhaustion score and cynicism score resulted in similar results. We used the binary definition here to reduce the number of tables.

^b^ All the satisfaction and agreement factors were specified as binary variable where very satisfied and satisfied (strongly agree and agree) are coded as 1 and everything else as 0.

According to the 2017 survey, compared with physicians in midcareer (11-20 years since training), early-career physicians (≤10 years since training) had an increased association with burnout (OR, 1.36; 95% CI, 1.05-1.77), while physicians in their late career (>30 years since training) had a decreased association (OR, 0.59; 95% CI, 0.40-0.88). Similarly, physicians who had completed training in the previous 21 to 30 years had a decreased association (OR, 0.84; 95% CI, 0.61-1.16). Among specialties, primary care physicians had an increased association with burnout (OR, 1.42; 95% CI, 1.02-1.99). Among work-related factors, satisfaction with workflow, relationship with colleagues, time and resources for continuing medical education, opportunity to affect decision making, workload, and having a trusted advisor were associated with low likelihood of burnout.

In both surveys, physicians were asked to indicate their plan for the next 3 years. Table 4 presents the association of emotional exhaustion and physicians’ plan. We first sorted physicians into quartiles of emotional exhaustion (from lowest to highest) and then calculated the percentage of physicians who planned to continue in their current role as well as those who planned to make changes. In the 2017 survey, relative to physicians in the first quartile, physicians in the fourth quartile were less likely to continue in their current role (80.8% vs 44.4%), more likely to cut their work hours (9.0% vs 31.5%), more likely to decrease the number of patients in their practice (5.2% vs 20.2%), and more likely to relocate to another practice (3.2% vs 17.4%). Aspirations to attain an administrative role in the organization and to retire from practice were less associated with level of exhaustion. In multivariate analysis and after controlling for basic demographic characteristics, exhaustion was associated with several outcomes, but not with intent to seek an administrative position within the organization (Table 4). A 1-unit increase in exhaustion (scale of 0-6) was associated with 38% decrease in likelihood of continuing in current role (95% CI, 42%-33%), 58% increase in likelihood of reducing work hours (95% CI, 44%-73%), 48% increase in likelihood of reducing number of patients in practice (95% CI, 32%-65%), 61% increase in likelihood of relocating to other practice (95% CI, 42%-83%), and 38% increase in likelihood of retiring (95% CI, 15%-66%). The results were similar for the 2014 survey.

**Table 4.** Effect of Exhaustion on Future Plan

Plan for Next 3 y	Full Sample, No. (%)	Respondents in Each Q of Exhaustion (Lowest to Highest), %				Multivariate Analysis (Effect of 1-Unit Increase in Exhaustion) Odds Ratio (95% CI)^a^
		Q1	Q2	Q3	Q4	
**2017 Survey**						
Continue current role	1207 (64.1)	80.8	69.3	60.5	44.4	0.62 (0.58-0.67)
Decrease No. of work hours	360 (19.1)	9.0	13.6	23.5	31.5	1.58 (1.44-1.73)
Decrease No. of patients in practice	225 (12.0)	5.2	9.1	14.0	20.2	1.48 (1.32-1.65)
Relocate to another practice	166 (8.8)	3.2	7.0	8.1	17.4	1.61 (1.42-1.83)
Seek administrative role	170 (9.0)	7.2	9.1	8.6	11.4	1.12 (1.00-1.25)
Retire	68 (3.6)	3.0	3.4	3.6	4.5	1.38 (1.15-1.66)
**2014 Survey**						
Continue current role	1161 (65.5)	77.6	74.8	62.1	45.6	0.67 (0.63-0.72)
Decrease No. of work hours	320 (18.1)	9.7	13.2	19.5	19.5	1.48 (1.36-1.62)
Decrease No. of patients in practice	213 (12.0)	5.9	8.2	12.0	22.7	1.43 (1.29-1.59)
Relocate to another practice	162 (9.1)	3.8	7.3	11.3	14.8	1.48 (1.32-1.67)
Seek administrative role	172 (9.7)	7.6	7.5	12.0	12.0	1.14 (1.02-1.26)
Retire	65 (3.7)	5.3	2.0	2.8	4.4	1.06 (0.89-1.25)

Abbreviation: Q, quartile.

^a^ Logistic regression model was specified for each outcome (plan for next 3 years) and adjusted for sex, race, years since training, specialty, and exhaustion score.

## Discussion

Similar to other studies, our survey shows an increase in physician burnout rate over time. The higher burnout rate documented in 2017 may be consequent to the implementation of a new electronic health record (EHR) across the institution. Average time spent on administrative tasks increased from 23.7% in 2014 to 27.9% in 2017, and time spent on administrative tasks was positively associated with higher likelihood of burnout in both the 2014 and 2017 surveys. Others have documented similar findings. In a national study^23^ of 6375 physicians, of whom 5389 (84.5%) used an EHR platform, lower rates of satisfaction with the amount of time spent on clerical tasks and higher burnout rates were noted. Machine learning and machine intelligence algorithms may offer an opportunity for consolidation of information in the EHR and provide relevant summary of these data, sparing clinicians this clerical role. The McKinsey Global Institute^24^ projects a 30% improvement in nursing productivity from adoption of these technologies.

The increasing burnout rate seen in our study over time may also be the result of an increasing mismatch between physicians’ interests within medicine and the growing demands placed on them that subtract meaning and joy from practice. Other studies support our results. Increasing administrative requirements, loss of autonomy, exhaustion, practice setting and resources, and choice of specialty have been implicated as factors contributing to physician burnout.^8,11,18^ Medicine can be a decidedly technical and intellectually challenging profession, characterized by a continuous demand in the execution of high-stakes decisions that often require judgement and lack certainty. Relationships with patients and meaningful intellectual clinical, research, and educational work mitigate the challenges of this demanding charge.^8^ The associations of numerous factors with burnout and the individual physician are complex and dynamic and change over time and based on the work environment.

In our study, we found physicians were more vulnerable to emotional exhaustion than any of the other subscales of burnout. Physicians reporting high levels of exhaustion were more likely to reduce their clinical schedules, reduce the number of patients in their practice, leave the practice, or retire. The association between burnout and physician clinical work effort, as well as turnover, has been described extensively in the literature.^25,26,27,28,29,30^ In a longitudinal prospective study^30^ evaluating the association between burnout and physician work effort, the authors reported that for every 1-point decrease in satisfaction, there was a 30% to 40% increase in the likelihood that a physician would decrease work effort over the subsequent 2 years.

Physician turnover is associated with patient and clinician distress, as well as financial cost to the system. Studies show that the cost of replacing a physician, irrespective of practice, is 2 to 3 times the individual’s annual salary.^25,26,27,28,29^ Primary care physicians in our survey were more likely to report high levels of exhaustion when compared with medical specialists, suggesting they represent a population requiring targeted interventions to address exhaustion. These findings may be associated with the amount of time primary care physicians spend documenting on the EHR and serving as the clinicians responsible for the management of patients’ multiple complex medical and social problems.^23^

After adjusting for potential relevant factors, we found physicians with more than 30 years of experience demonstrated lower rates of burnout, while early-career physicians (<10 years in practice) demonstrated higher rates of burnout. Other studies in the peer-reviewed literature have demonstrated younger age to be independently associated with higher rates of burnout.^8,31^ In multivariate analysis, factors associated with burnout in the early-career group included respondents’ satisfaction with department leadership, relationship with colleagues, quality of clinical care delivered, control over work environment, and poor career fit (spending <20% of time on an activity, such as research, teaching, patient care, or administration, that the physician finds most meaningful).^32^ These findings point to potential opportunities in this vulnerable group to mitigate burnout, such as initiatives that promote community building and networking and harnessing effective leadership.^8^ In a study^33^ including 2813 physicians, after adjusting for relevant variables, each 1-point increase in leadership score was associated with a concomitant 3.3% decrease in burnout and 9% increase in career satisfaction (*P* < .001).

In multivariate analysis, satisfaction with workload and relationship with colleagues were associated with lower burnout rates, while more time spent on administrative duties and lack of control over one’s schedule had inverse associations. Other studies have shown that these factors are associated with burnout.^30,34^ Continued decline in reimbursements coupled with increased expenditure in health care systems, such as investments in EHR platforms, have resulted in significant capital expenditures. These demands have been addressed by health care organizations, at least in part, by placing increasing productivity and higher efficiency demands on physicians and reducing health care delivery cost.^25^ Furthermore, the health care industry is faced with larger demands for implementation, measurement, and reporting of quality metrics, for example, and growing assessment centered on reporting patient satisfaction and experience.^25^ The end result has been increasing administrative tasks as part of physicians’ daily responsibilities, often accompanied by allocation of fewer resources. Each of the burnout domains identified in this study is influenced by the individual physician, practice, organization, and national health care system. Addressing factors associated with burnout identified in our study and extensively documented in the literature will necessitate a shared commitment from both health care organizations and individual physicians.^25,34^ Furthermore, efforts to enhance and maintain connectivity among physicians are also critical.

### Limitations

Our study has several limitations. Our findings may not be generalizable to the physician population at large because the study was conducted in a single academic medical center. Although all respondents spend some time delivering clinical care, many of them also spend significant portions of their time engaged in research, education, and other administrative roles. Another study limitation is our classification of burnout into a dichotomous variable rather than a continuum of low to high with reported cutoff values for each classification. The result of our applied methodology may be an overestimate of burnout rates.^35^ When we evaluate our data using the continuum methodology, we observed a proportional increase in burnout between the 2 points comparable to the results using a dichotomous variable. The anonymous nature of the study is another limitation. For example, we do not have data on specific actions physicians intending to address their burnout undertook, or the specific effect exhaustion had on their practice. Study strengths include a robust survey response across a large multispecialty academic medicine practice. Furthermore, our survey included physicians from multiple specialty disciplines, practice settings, and environments.

## Conclusions

Remediation of burnout in health care necessitates centrally and locally designed initiatives. Efforts will also require attention, recognition, and accountability from executive and regional institutional leadership. Solutions to address physician burnout will entail shared commitment from physicians and organizations, as well as physician-, practice-, and institution-level initiatives.
